# Efficacy of combination of N-acetylcysteine and primrose in spinal cord injury; an experimental study

**DOI:** 10.1016/j.heliyon.2023.e19350

**Published:** 2023-08-21

**Authors:** Umut Yücel Çavuş, Abdurrahman Yılmaz, Mustafa Begenc Tascanov, Metin Ocak

**Affiliations:** a University of Health Sciences DıskapıYıldırım Beyazıt Education and Training Hospital, Department of Emergency Medicine,Ankara, Turkiye; b Uşak University Faculty of Medicine, Department of Emergency Medicine, Uşak, Turkiye; c Department of Cardiology, Harran University Faculty of Medicine, Sanliurfa, Turkiye; d Samsun Education and Training Hospital, Department of Emergency Medicine, Samsun, Turkiye

**Keywords:** Spinal cord injury, N-Acetylcysteine, primrose, Secondary injury

## Abstract

**Introduction:**

Spinal cord trauma represents a major cause of emergency department admissions, with high morbidity and mortality rates. It requires early and urgent treatment. This experimental study assessed the effectiveness of a combination of primrose and N-acetylcysteine (NAC) in managing spinal cord injury (SCI).

**Methods:**

We divided 46 adult male Wistar albino rats (6–8 months old, weighing 300–350 g) into five groups. Group 1 (n = 10) received only primrose; group 2 (n = 10) received only NAC; group 3 (n = 10) received a combination of NAC and primrose; group 4 (n = 10) received no intervention (first control group); group 5 (n = 10) underwent laminectomy only (second control group). Intergroup neurological and motor function were evaluated on days 1, 7, and 14. Oxidative biochemical markers, such as superoxide dismutase (SOD), glutathione peroxidase (GPX), and malondialdehyde (MDA), were measured.

**Results:**

Significant differences were recorded in the GPX, SOD, and MDA values of groups 1, 2, 3, and 4 (p < 0.001, p = 0.005, and p = 0.097, respectively). Groupwise comparisons were conducted to identify the clinical significance of these markers. GPX and SOD levels were significantly higher in group 1 than in group 2; MDA levels were lower in group 1. GPX and SOD levels were significantly higher than in group 3 than in group 1; MDA levels were lower in group 3. Compared with group 5, group 1 demonstrated significantly higher GPX and SOD levels and lower MDA levels. Results in group 2 were similar to results in group 5. In group 3, GPX and SOD levels were significantly higher than in groups 2 and 5; MDA levels were significantly lower. Comparisons according to inclined plane angle level and motor function values revealed significant results on day 14, in favor of group 3 rats that had received the combined treatment.

**Conclusion:**

The combined administration of NAC and primrose for traumatic SCI was more effective than either treatment alone in terms of improving biochemical and neurological functions. These findings suggest that the combination of NAC and primrose can serve as an effective treatment option for traumatic SCI.

## 1 Introduction

Spinal cord trauma is a major cause of emergency department admissions because of its high morbidity and mortality rates. Multiple trauma factors (e.g., traffic accidents and falls from a height) frequently result in spinal cord injury (SCI), primarily among young people. Some studies have revealed an SCI mortality rate of approximately 50% [1].

Traumatic SCI occurs through two main mechanisms. The primary injury leads to necrotic cell death; within hours, secondary injuries influence biochemical and metabolic processes [2,3]. Studies exploring the physiopathology of SCI have revealed that edema, free radicals, and lipid peroxidation have key roles in the development of secondary injury. Additionally, the levels of antioxidants (e.g., glutathione peroxidase [GPX] and superoxide dismutase [SOD]) and the oxidant malondialdehyde (MDA) could serve as indicators for SCI progression [4,5].

There is a substantial need for medical treatment of SCI in a manner that demonstrates curative efficacy and has a low risk of side effects. Numerous antioxidants have been tested, and many others are under investigation. High-dose methylprednisolone, previously used in emergency treatment of secondary SCI damage, is no longer recommended because of potential side effects; thus, the search for alternative therapies continues [4].

N-acetylcysteine (NAC) is a glutathione precursor that inhibits the formation of free radicals. Previous studies have demonstrated that NAC has a protective effect on traumatic SCI [4,5]. Primrose, a member of the Onagraceae family and genus Oenothera, has various pharmacological effects, including antimicrobial and antioxidant properties. Oils and extracts from primrose are traditionally used in skin treatments; they can also serve as natural antioxidants [[6], [7], [8]].

This study was performed to compare the efficacy of high-dose NAC and primrose in an experimental rat model of traumatic SCI. We present the neuroclinical and biochemical results of combining these two antioxidants.

## 2 Materials and methods

The experimental protocol adhered to the "Guide for the Care and Use of Laboratory Animals" and was approved by the ethics review committee of the University of Health Sciences. All animal rights were respected throughout the study. In total, 46 adult male Wistar albino rats (aged 6–8 months, weighing 300–350 g) were divided into five groups. The rats were housed at room temperature (22°C–25 °C) with free access to food and water. They were kept in a veterinary laboratory under favorable conditions for 14 days. All rats were examined prior to the experiment, and only rats with normal neurological function were included in the study.

Among the five groups, two were designated as control groups and three were designated as treatment groups. Group 1 (n = 10) received SCI + primrose; group 2 (n = 10) received SCI + NAC; group 3 (n = 10) received SCI + combined NAC/primrose treatment; group 4 (n = 10) received no intervention (first control group); and group 5 (n = 10) received SCI + 1 mL of intraperitoneal saline in bolus form (second control group).

**Fig. 1:**
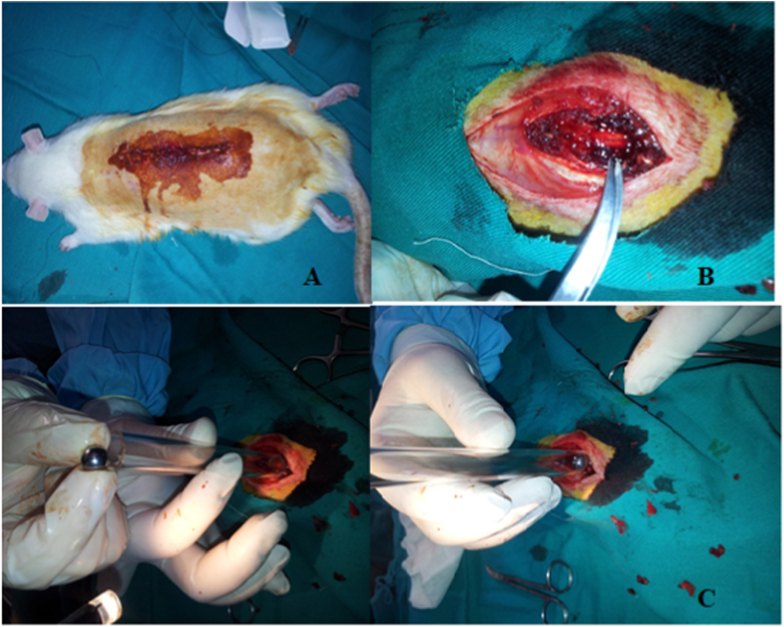
**A:** Postoperative rat. **B:** Exposure of the spinal cord after laminectomy. **C:** Modified Allen's weight-drop model.

All groups, except group 4, received intraperitoneal ketamine hydrochloride 50 mg/kg (Istanbul, Turkey) and xylazine 10 mg/kg (Bayer, Germany) for anesthesia. Each rat was secured in the prone position on a board. The thoracic area was sterilized with 10% polyvinylpyrrolidone iodine, and the fur was shorn. The area was re-sterilized after shearing. A 3-cm incision was made at the T5–T12 level with respect to the interscapular area; the subcutaneous tissues and paravertebral muscles were retracted. The laminae were exposed, and a laminectomy was performed at the T7–T9 level (Fig. 1A–B).

A modified Allen weight-drop model was used to induce trauma (Fig. 1 C) [9,10]. For the rats undergoing trauma, the dura mater was resected intact under sterile surgical conditions. A glass pipe, with a diameter of 0.5 cm and a length of 12.2 cm, served as a guide for a sterile ball weighing 4.1 g. The ball was dropped at the T7–T9 level, subjecting the rats to an impact of 50 g/cm on the dorsal surface of the spinal cord. After hemostasis was achieved with a bipolar coagulator, the paravertebral muscles and skin were sutured using 4/0 VICRYL®. The rats were allowed to awaken at room temperature, and the development of paraplegia was tested by a painful stimulus to the tail. Paraplegia was observed in all operated rats.

Drug Management: Primrose at a dosage of 6000 mg/day was administered to rats in group 1 through an esophageal feeding tube [11,12]. Rats in group 2 received a 300 mg/kg intraperitoneal bolus of NAC [4,5]. Rats in group 3 simultaneously received primrose (6000 mg/day through an esophageal feeding tube) and an intraperitoneal bolus of NAC (300 mg/kg). Treatments for all groups were initiated within the first hour after trauma.

**Fig. 2:**
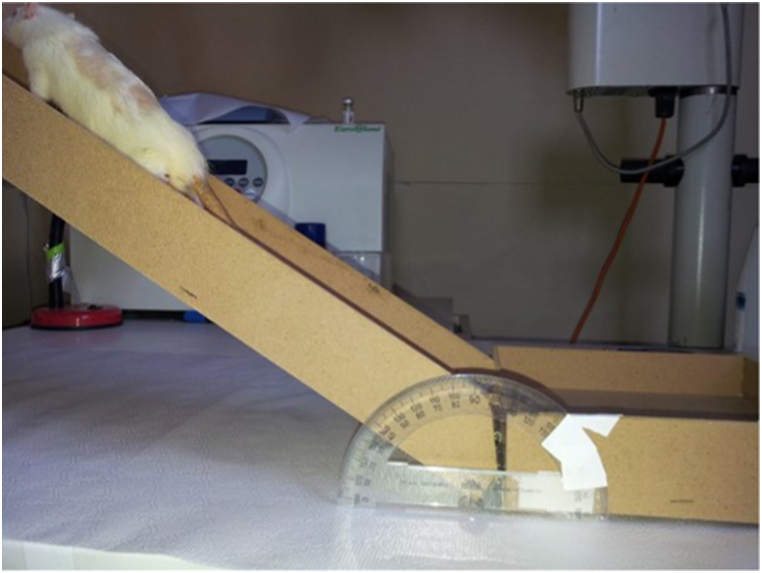
Inclined plane test (Rivlin–Tator).

Neuroclinical Examination: Motor functions were assessed on the 1st, 7th, and 14th days using the inclined plane test and the motor scale [13,14] (see Fig. 2 and Table 1). An evaluator, blinded to the group assignments, observed the rats on inclined and flat planes. Functional improvement was determined by.

(a) Inclined Plane Test: Rats were positioned on a flat board aligned with the ground. The board was elevated from one edge, and the tilt was progressively increased. The highest angle at which a rat could maintain a stable position without falling for 5 s was recorded as the inclined plane angle. This test was performed on the 1st, 7th, and 14th days after surgery.
(b) Motor Function Assessment: Regular motor evaluations were conducted to monitor functional changes. Motor function was assessed on the 1st, 7th, and 14th days after surgery.
Biochemical Evaluation: Spinal cord samples from the lesion site were extracted under general anesthesia and sterile conditions on the 14th day for biochemical analysis. Samples of approximately 110 mg, harvested from each rat, were stored under suitable conditions and frozen at −80 °C. The rats were then euthanized by aspiration of cardiac blood under anesthesia.

**Table 1:** Comparison of biochemical values between groups.

	Group 1 (n:10)	Group 2 (n:10)	Group 3 (n:10)	Group 4 (n:10)	P Values
**GPX**	182.09 ± 24.32	170.12 ± 41.29	588.62 ± 324.65	652.50 ± 150.33	0.000
**SOD**	109.04 ± 116.37	74.07 ± 57.83	292.86 ± 253.24	70.13 ± 12.62	0.005
**MDA**	86.91 ± 44.47	104.85 ± 54.02	56.48 ± 25.47	77.39 ± 25.45	0.097

**Abbreviations:** GPX: glutathione peroxidase, SOD: superoxide dismutase, MDA: malondialdehyde, Group 1 = SCI + primrose; Group 2 = SCI + NAC; Group 3 = SCI + combined NAC/primrose treatment; Group 4 = no intervention.

Protein Homogenization and Determination of Protein Content: Spinal cord tissue samples from each rat were weighed prior to the preparation of supernatants and homogenates, in accordance with the technique established by Irmak et al. Protein measurements were conducted using the method developed by Lowry et al. with commercial chemicals and reagents supplied by Sigma (MO, USA) [15,16].

Determination of MDA Content: The MDA content in spinal cord tissue was measured by spectrophotometry, based on the reaction of MDA with thiobarbituric acid to produce a pink pigment with maximum absorption at 532 nm. After the addition of thiobarbituric acid to the supernatant, the mixture was heated to 90–100 °C for 15 min at a pH of 2–3. This was followed by the addition of two volumes of cold 10% (w/v) trichloroacetic acid to precipitate the protein. After centrifugation, the supernatant was mixed with an equal volume of 0.67% (w/v) thiobarbituric acid in a boiling water bath for 10 min. After the mixture had been cooled, its absorbance was measured at 532 nm [17].

Determination of GPX and SOD Levels: The levels of GPX and SOD activity in spinal cord tissues were evaluated spectrophotometrically using a commercial assay kit from Cayman.

## 3 Statistical analyses

Data analysis was conducted using IBM SPSS Statistics version 25.0 software (IBM Corporation, NY, USA). The Kolmogorov–Smirnov test was utilized to examine data normality. Descriptive statistics were expressed as either means ± standard deviations or medians (25th–75th percentiles), as applicable. Student's t-test was used for comparisons of two groups; one-way analysis of variance was used for comparisons of three groups. P-values <0.05 were considered statistically significant.

## 4 Results

The study involved five distinct groups, including two control and three treatment groups. For biochemical evaluation, antioxidant parameters (GPX and SOD levels) and an oxidant parameter (MDA level) were measured. Groups 4 and 5 were compared to establish baseline values of these parameters: GPX (652.50 ± 150.33 vs. 474.75 ± 138.88, P = 0.013), SOD (70.13 ± 12.62 vs. 55.06 ± 8.89, P = 0.006), and MDA (77.39 ± 25.45 vs. 173.94 ± 98.71, P = 0.008) (Table 2). GPX, SOD, and MDA exhibited significant differences (p < 0.001, p = 0.005, and p = 0.097, respectively) among the five groups (Table 2).

**Table 2:** Comparison of biochemical markers between groups.

Compared Groups	GPX	SOD	MDA
Group 4 (n:10)	652.50 ± 150.33	70.13 ± 12.62	77.39 ± 25.45
Group 5 (n:10)	474.75 ± 138.88	55.06 ± 8.89	173.94 ± 98.71
P Values	0.013	0.006	0.008
Group 1 (n:10)	182.09 ± 24.32	109.04 ± 116.37	86.91 ± 44.47
Group 2 (n:10)	170.12 ± 41.29	74.07 ± 57.83	104.85 ± 54.02
P Values	0.043	0.031	0.876
Group 1 (n:10)	182.09 ± 24.32	109.04 ± 116.37	86.91 ± 44.47
Group 3 (n:10)	588.62 ± 324.65	292.86 ± 253.24	56.48 ± 25.47
P Values	0.004	0.000	0.445
Group 2 (n:10)	170.12 ± 41.29	74.07 ± 57.83	104.85 ± 54.02
Group 3 (n:10)	588.62 ± 324.65	292.86 ± 253.24	56.48 ± 25.47
P Values	0.003	0.000	0.436
Group 1 (n:10)	182.09 ± 24.32	109.04 ± 116.37	86.91 ± 44.47
Group 5 (n:10)	126.00 ± 122.52	55.06 ± 8.89	173.94 ± 98.71
P Values	0.002	0.000	0.011
Group 2 (n:10)	170.12 ± 41.29	74.07 ± 57.83	104.85 ± 54.02
Group 5 (n:10)	126.00 ± 122.52	55.06 ± 8.89	173.94 ± 98.71
P Values	0.004	0.001	0.016
Group 3 (n:10)	588.62 ± 324.65	292.86 ± 253.24	56.48 ± 25.47
Group 5 (n:10)	126.00 ± 122.52	55.06 ± 8.89	173.94 ± 98.71
P Values	0.033	0.001	0.001

**Abbreviations:** GPX: glutathione peroxidase, SOD: superoxide dismutase, MDA: malondialdehyde; Group 1 = SCI + primrose; Group 2 = SCI + NAC; Group 3 = SCI + combined NAC/primrose reatment; Group 4 = no intervention; Group 5 = SCI + intraperitoneal saline.

Next, the clinical implications of these markers were analyzed by pairwise comparisons (Table 3). Comparisons of groups 1 and 2 revealed that GPX and SOD levels were significantly higher, and MDA levels were lower, in group 1. Comparisons of groups 1 and 3 revealed that GPX and SOD levels were significantly higher, and MDA levels were significantly lower, in group 3. There were similar trends in comparisons of groups 1 and 5 and groups 2 and 5. Comparisons of groups 2 and 3 revealed that GPX and SOD levels were significantly higher in group 3; MDA levels were not statistically significantly different but tended to be lower in group 3.

**Table 3:** Comparison of motor function and inclined plane angle between groups.

Motor Function Values	Group 1 (n:10)	Group 2 (n:10)	Group 3 (n:10)	P Values
1st day	3.8 ± 0.17	3.7 ± 0.48	3.8 ± 0.8	0.934
7th day	4.3 ± 0.44	3.6 ± 0.80	4.8 ± 0.58	0.072
14th day	4.8 ± 0.58	4.3 ± 0.70	4.9 ± 0.8	0.006
**Inclined Plane Angle Values**	**Group 1 (n:10)**	**Group 2 (n:10)**	**Group 3 (n:10)**	**P Values**
1st day	38.8 ± 2.8	39.3 ± 3.6	38.6 ± 5.5	0.806
7th day	41.6 ± 3.3	41.5 ± 2.9	44.6 ± 2.6	0.006
14th day	42.8 ± 1.9	40.6 ± 1.7	46.9 ± 3.7	0.050

**Abbreviations:** Group 1 = SCI + primrose; Group 2 = SCI + NAC; Group 3 = SCI + combined NAC/primrose treatment.

Groups were also compared according to inclined plane test angle levels and motor function scores (Table 4). Statistically significant differences among groups were observed on the 7th and 14th days for motor function scores, but not on the 1st day. In terms of angle levels, a statistically significant difference was observed on the 14th day, but not on the 1st and 7th days.

**Table 4:** Comparison of groups according to motor function and inclined plane test angle.

Motor Function	Day 1	Day 7	Day 14
Group 1 (n:10)	3.77 ± 0.17	4.30 ± 0.44	4.70 ± 0.53
Group 2 (n:10)	3.70 ± 0.48	3.60 ± 0.80	4.33 ± 0.70
P Values	0.683	0.044	0.253
Group 1(n:10)	3.77 ± 0.17	4.30 ± 0.44	4.70 ± 0.53
Group 3 (n:10)	3.87 ± 0.83	4.87 ± 0.58	4.91 ± 0.15
P Values	0.200	0.043	0.138
Group 2 (n:10)	3.70 ± 0.48	3.60 ± 0.80	4.33 ± 0.70
Group 3 (n:10)	3.87 ± 0.83	4.87 ± 0.58	4.90 ± 0.10
P Values	0.828	0.002	0.018
**Inclined Plane Test**	**Day 1**	**Day 7**	**Day 14**
Group 1 (n:10)	38.77 ± 2.87	41.63 ± 3.34	42.75 ± 1.94
Group 2 (n:10)	39.30 ± 3.59	41.50 ± 2.95	40.55 ± 1.66
P Values	0.742	0.927	0.024
Group 1 (n:10)	38.77 ± 2.87	41.63 ± 3.34	42.75 ± 1.94
Group 3 (n:10)	38.62 ± 5.52	44.62 ± 2.55	46.87 ± 3.72
P Values	0.947	0.065	0.015
Group 2 (n:10)	39.30 ± 3.59	41.50 ± 2.95	40.55 ± 1.66
Group 3 (n:10)	38.62 ± 5.52	44.62 ± 2.55	46.87 ± 3.72
P Values	0.758	0.031	0.001

**Abbreviations:** Group 1 = SCI + primrose; Group 2 = SCI + NAC; Group 3 = SCI + combined NAC/primrose treatment.

For a more detailed understanding of clinical implications, pairwise comparisons of inclined plane test angle levels and motor function scores were conducted (Table 4). Comparisons of groups 1 and 2 revealed that motor function scores were significantly higher in group 1 on the 7th and 14th days; motor function scores did not significantly differ on the 1st day. Comparisons of groups 1 and 3 revealed significantly higher motor function scores on the 14th day in group 3. Similarly, comparisons of groups 2 and 3 revealed significantly higher motor function scores on the 14th day in group 3.

There were no significant differences in inclined plane test angle between group 1 and group 2. Comparisons of groups 1 and 3, as well as groups 2 and 3, revealed significantly higher inclined plane test angles on the 14th day in group 3.

## 5 Discussion

This experimental study demonstrated that the combined administration of primrose and NAC in rats with traumatic SCI significantly improved antioxidant biochemical markers, motor function, and inclined plane angle values by the 14th day. To our knowledge, this is the first investigation of the combined effects of NAC and primrose on traumatic SCI.

Traumatic SCI comprises primary damage, which occurs as an immediate result of trauma, and secondary damage, which occurs gradually because of mechanisms triggered by the primary damage. Because secondary damage amplifies mortality and morbidity, early interventions to mitigate its effects are critical. Consequently, research efforts have been focused on preventing secondary damage [[3], [4], [5],^18^]. Secondary damage arises from a progressive cascade of local tissue deterioration, encompassing changes in the microenvironment surrounding the damaged spinal cord (e.g., inflammation, edema, microcirculatory ischemia, and apoptosis) [19]. Accordingly, treatments for SCI primarily focus on managing inflammation, oxidative stress, and apoptosis, which constitute secondary injuries. The elimination of free radicals is suspected to prevent or lessen neurological deficits and damage [5]. Despite extensive use of high-dose methylprednisolone to hinder secondary damage in traumatic SCI, its potential side effects have led to a search for more reliable and effective treatment methods [4].

Evening primrose oils and extracts have been used as natural antioxidants. The predominant components of primrose are phenol compounds, which confer its antioxidant and free radical scavenging properties. Primrose reportedly contains 9.24% gamma-linolenic acid, 73.88% linoleic acid, and 6.93% oleic acid, along with other components [7]. Gamma-linolenic acid in primrose is regarded as an anti-inflammatory eicosanoid precursor. There is evidence that primrose's ethanolic extracts exhibit antioxidant effects [20], and its methanolic extracts potentially have antioxidant properties [21]. Previous research suggested that primrose could reverse neuro-conduction disorders in diabetic rats [22]; halt Alzheimer's progression in rats; and significantly enhance cognitive function, vitality, and quality of life in patients with multiple sclerosis [7,8]. To our knowledge, the present study is the first to examine the effects of primrose on SCI. We discovered that primrose alone was more effective than NAC alone in terms of oxidative biochemical markers in rats with traumatic SCI, although it was less effective than the combined use of NAC and primrose.

Endogenous glutathione has a robust inhibitory effect on secondary injury in experimental SCI. NAC, a precursor to glutathione, is a neuroprotective agent often recognized for its potent antioxidant capabilities [1,23]. Lin et al. demonstrated that NAC provides a neuroprotective effect in cortical neuron cultures by influencing genes such as p42 and p44 extracellular signal regulatory kinase (ERK), p38 mitogen-activator protein kinase (MAPK), and p53 [24]. Numerous previous studies have shown that NAC treatment can prevent or diminish secondary damage in SCI; it may be a useful therapeutic agent because of its neuroprotective effects [1,4,5,18,19,[23], [24], [25], [26]]. In our study, consistent with published literature, we observed a significant difference (in terms of oxidative biochemical markers in rats with traumatic SCI) in the group administered NAC alone, compared with the control group. Nevertheless, this positive effect was significantly smaller than the effect of primrose alone or a combination of NAC and primrose.

Previous studies have indicated that the combined use of two agents or parameters has a more robust therapeutic effect and predictive value, compared with the use of any single parameter [1,26]. In the present study, we found that the combined use of primrose and NAC was more effective than either of these treatments alone in terms of oxidative biochemical markers, neurological function, and motor function. These findings support our hypothesis that a combination of primrose and NAC has strong therapeutic effects.

## 6 Limitations

Considering the sample size estimates, the number of animals in this study was relatively small. Moreover, we were unable to conduct NAC infusion therapy or perform histopathological examinations. Because this was an experimental study, the findings may lack broad generalizability.

## 7 Conclusions

Our experimental study findings indicate that the combined application of NAC and primrose in traumatic SCI is more effective than either treatment alone in terms of biochemical markers and neurological function. Our results suggest that a combination of NAC and primrose can serve as an effective treatment option in traumatic SCI.

## Author contributions

1 - Conceived and designed the experiments: U.Y.C; A.Y.; M.O; and M.B.T., 2 - Performed the experiments; U.Y.C; A.Y.; M.O; and M.B.T. 3 - Analyzed and interpreted the data: U.Y.C; A.Y.; M.O; and M.B.T., 4 - Contributed reagents, materials, analysis tools or data: U.Y.C; A.Y.; M.O; and M.B.T.,. 5 - Wrote the paper. M.B.T., M.O. and U.Y.C.

## Funding

There was no funding provided for this article.

## Declaration of competing interest

The authors declare that they have no known competing financial interests or personal relationships that could have appeared to influence the work reported in this paper.
